# Cost-effectiveness of home care services versus hospital care for pediatric patients worldwide: A protocol for systematic review and meta-analysis

**DOI:** 10.1097/MD.0000000000030993

**Published:** 2022-10-14

**Authors:** Luís Carlos Lopes-Júnior, Raphael Manhães Pessanha, Emiliana Bomfim, Regina Aparecida Garcia de Lima

**Affiliations:** a Graduate Program in Public Health, Health Sciences Center at the Federal University of Espírito Santo (UFES), Vitória, ES, Brazil; b McGill University. Montreal, Quebec, Canada; c University of São Paulo. Ribeirão Preto, SP, Brazil.

**Keywords:** cost-effectiveness, health systems, home care, public health, systematic review

## Abstract

Despite the expansion of home care services (HCS) in several countries, there is still a need to systematically investigate the available evidence on the cost-effectiveness of this type of service compared to hospital care in the world, particularly for the pediatric population. Hence, we aimed to systematically synthesize and critically evaluate the evidence on the cost-effectiveness of HCS versus in-hospital services worldwide.

**Methods:**

A systematic review and meta-analysis protocol guided by Preferred Reporting Items for Systematic Reviews and Meta-Analyses Protocols. Ten databases will be searched: MEDLINE/PubMed, Cochrane Library, Excerpta Medica database, cummulative index to nursing and allied health literature (CINAHL), Web of Science, SCOPUS, Science Direct, PsycINFO, Latin American and Caribbean Health Sciences Literature and Chinese national knowledge infrastructure with no restrictions on publication date or languages. A checklist for assessing the quality of reporting of economic evaluation studies will be applied. To assess the methodological quality of evidence from observational research on comparative effectiveness, the Good Research for Comparative Effectiveness Checklist v5.0 will be used. The heterogeneity among the studies will be assessed using the *I*^2^ statistic test. According to the results of this test, we will verify whether a meta-analysis is feasible. If feasibility is confirmed, a random-effect model analysis will be carried out. For data analysis, the calculation of the pooled effect estimates will consider a 95% CI and alpha will be set in 0.05 using the R statistical software, v.4.0.4. In addition, we will rate the certainty of evidence based on Grading of Recommendations Assessment, Development and Evaluation. All methodological steps of this review will be performed independently and paired by 2 reviewers and conducted and managed in the EPPI-Reviewer Software™.

**Results:**

The results may have relevance for the basis of public health policies, regarding the forms of organization of HCS, especially in terms of complete economic evaluations through cost-effectiveness analysis in relation to hospital care.

**Conclusion:**

To the best of our knowledge this will be the first systematic review and metanalysis to synthesize and critically evaluate the evidence on the cost-effectiveness of HCS versus in-hospital services worldwide. The review will adopt a rigorous approach, adhering to PRISMA Statement 2020, using a comprehensive and systematic search strategy in 10 databases, further the gray literature, pre-prints, with no time period or language restrictions.

## 1. Introduction

Worldwide, Synchronously, the changes in the demographic and epidemiological transition that are taking place in most countries regardless of income level, pointing to a situation of triple burden of disease with the hegemonic presence of chronic conditions,^[1]^ the need to adapt the health care model has emerged, leading many countries to think of Home Care Services (HCS) as a strategic point of care for health care.^[2]^ The increase of HCS in several countries follows, in parallel, the interest of health systems in the process of de-hospitalization, rationalization of the use of hospital beds, cost reduction and organization of patient-centered care.

The demand for HCS appears, therefore, as another challenge for health systems, contributing to change the focus of care and the environment in which care is provided.^[2,3]^ In addition, continued care in the home environment is accompanied by other equally relevant health needs, such as the aging population, as well as the care provided to premature babies, to children with special needs and chronic diseases, to adults with multiple chronic-degenerative diseases, to individuals in palliative care, life support and rehabilitation.

Thus, the relevance of the need to implement HCS stands out in the current and future health agenda of all health systems, aiming to contribute to the configuration of substitutive health networks and the transformation of health practice.^[2–4]^ Researchers already question the real need for hospitalization for certain health problems, considering some reasons for hospitalization as dispensable or unnecessarily prolonged, and that they can be replaced or complemented by an HCS.^[2–5]^ In addition to cost reduction, the HCS has been representing in the world scenario the connotation of offering quality care, providing welfare and comfort by allowing the patient to stay in their home environment, integrated into their life context.^[5]^

It is important to emphasize that health financing is a topic that always raises debates in order to better define the use and allocation of resources for society as a whole. Most countries face increasing health care costs, both in absolute and relative terms, regardless of whether the financing model adopted is public, private, based on tax collection, or through direct user fees. The growth of health expenditures, with the need to seek efficiency in resource allocation, has occupied an important role in public policy discussions.^[6]^

Economic evaluation studies, such as those of cost-effectiveness, are adopted in order to consider the cost factor in decision-making regarding the new technologies or models of health care, since financial resources are scarce and finite. In the evaluation of health care models, cost-effectiveness analysis is the most suitable method to compare 2 or more alternatives because it allows the combined analysis of clinical benefits and associated costs, providing objective and explicit data for decision making.^[6]^ Cost-effectiveness studies can be understood as a tool to analyze the value of health interventions, since the method seeks to fill a gap between preferences and science. On one hand, there is the subjectivity of the preference that an individual or society has when faced with 2 options. On the other, there is the objectivity and the reproducibility of science, considering that the cost of a new technology or model of care needs to be manage.^[7]^ In cost-effectiveness evaluation, costs are confronted with clinical outcomes in an attempt to understand the impact of different alternatives, identifying those with better intervention effects, in general, in exchange for a lower cost to health systems.^[6–8]^

It is known that several countries are in the process of adapting to this new demand for care, due especially to issues of economic viability.^[2,3]^ This adaptation has strengthened the emergence of new strategies and mechanisms for health care, such as HCS, which combines technological and scientific resources present in the hospital with the family environment.^[9]^ The home has emerged, added to its humanizing characteristic and the demographic and epidemiological profile of the population, as a place with potential to expand and qualify the care processes.^[10]^ Moreover, the development of HCS on the world scene has been following demographic and epidemiological changes^[11]^ and is related to reducing the risk of infections^[12]^ the humanization of care and quality of life, greater involvement of family members with the patient’s illness, closer relationship between the health team, patient and family,^[13]^ cost reduction, increased hospital bed turnover with bed management, de-hospitalization^[14]^ lower rates of clinical worsening and acute complications, less demand for urgency and emergency services, and lower readmission rates,^[15]^ implementation of palliative care^[3]^ and effective actions for prevention, promotion and recovery of health.^[4]^

A systematic review and meta-analysis aimed at evaluating the effect of “hospital at home” services in adults >16 years that significantly replace inpatient time on health outcomes, showed that hospital at home is associated with reductions in mortality, re-admission rates and costs, and increases in patients and caregiver satisfaction, but no change in caregiver burden.^[16]^ Despite the expansion of HCS in several countries, there is still a need to systematically investigate the available evidence on the cost-effectiveness of this type of service in relation to hospital care in the world, particularly for the pediatric population.

Previous systematic review published in 2012 on the costs and effectiveness of pediatric home care pointed out that it can provide equivalent clinical outcomes for children and does not impose a greater burden on families.^[17]^ In fact, in some cases, there is evidence of reduced burden and costs to families compared to hospital care. There is also growing evidence, although based on weaker evidence, that pediatric home care can reduce health care costs, particularly for children with complex and long-term needs.^[18]^ It is noteworthy that this review was limited to the period from 1990 to 2007, and used simple methodological assessment tools available at the time.

Since there is an increasing use of decision analysis models in economic evaluations of technology-assisted care models, the use of specific methodological guidelines with the main aspects of modeling is necessary,^[18]^ since they can indicate how credible and relevant the result obtained is to inform decision makers. In this sense, systematic reviews of economic evaluations are important to synthesize the best evidence already available, in order to contribute to the formulation of guidelines for a new analysis, especially taking into account the most recent period (until 2022), in a context of contemporary transformations in health systems, and to identify the most relevant evaluations to inform a particular issue.^[19]^ Hence, the aim of this study is to systematically synthesize and critically evaluate the evidence on cost-effectiveness of home care versus in-hospital services in pediatric patients worldwide.

## 2. Methods

### 2.1. Study design

This is a systematic review and meta-analysis protocol guided by the Preferred Reporting Items for Systematic Reviews and Meta-Analyses Protocols.^[20]^ To ensure the reliability of the data and methodological transparency of this review, the protocol was submitted to the International Prospective Register of Systematic Reviews (NHS) for registration (Registration Number: CRD42022329687).

### 2.2. Research question

In order to formulate the research question, the PECOS strategy was used^[21]^ (P - Population or Patients; E - Exposure; C - Comparison; O - outcomes; S - study design), where P = Population = evidence of complete economic evaluations in pediatric patients, E = home care services, C = Comparison = hospital care (in-hospital services), O = Outcomes = mortality, readmission rates, patient and caregiver satisfaction, and cost; S = Study type: cost-effectiveness, cost-utility and cost-effectiveness evaluation studies.

In this study our population was stated in line with the World Health Organization definition, that is, “child” as a person under 19 years of age, an “adolescent” as a person aged 10 to 19 years, an “infant” as a person aged 0 to 11 months, and a “newborn” as a person aged 0 to 28 days.^[22]^ The acronym PECOS guided the structuring of the formulation of the research question: “What is the cost-effectiveness of home care compared to hospital care for pediatric patients in the world?"

### 2.3. Search strategy

The search for studies will be conducted systematically in ten electronic data-bases: Medical Literature Analysis and Retrieval System Online (MEDLINE) via PubMed, Excerpta Medica database, Cochrane Library, Web of Science, SCOPUS, Science Direct, Latin American and Caribbean Health Sciences Literature, Psychology Information (PsycINFO), cummulative index to nursing and allied health literature (CINAHL), and the Chinese National Knowledge Infrastructure. The strategy for seeking the studies will consist of a combination of controlled descriptors (indexers in the respective databases), synonyms, and keywords, as indicated in each electronic database. Thus, to search for articles in MEDLINE, we will use the Medical Subject Headings (MeSH) as controlled descriptors; the Emtree terms will be consulted in excerpta medica database; the PsycINFO Thesaurus will be consulted for the PsycINFO database; the DeCS (Health Sciences Descriptors) in the Latin American and Caribbean Health Sciences Literature database, and the CINAHL headings in the CINAHL database. It should be noted that there will be no date or language restriction in the search strategy to be performed. In addition to the electronic databases cited above, searches will be held on Clinical Trial Registry sites such as ClinicalTrials. gov (National Institutes of Health, NIH); World Health Organization International Clinical Trials Registry Platform, and the Brazilian Registry of Clinical Trials, as well as additional searches in sites of organizations and websites search, such as: The British Library, SciELO, Google Scholar, ProQuest Dissertations and Theses Global, Public Health Gray Literature Sources and Health Evidence and in the preprints for Health Sciences (medRxiv). Additionally, the list of final references from the included primary studies will be reviewed manually to find relevant studies to be added.

The search strategy will be carried out by 2 researchers independently (LCLJ and RMP) according to the recommendations of the Cochrane Handbook.^[23]^ Initially, the existence of an index of specific subject headings in each database (such as MeSH terms, Emtree terms, PsycINFO Thesaurus, DeCS and CINAHL headings and their synonyms and keywords will be identified. Subsequently, the search terms will be combined using the Boolean operators “AND” and “OR”^[24,25]^ in order to obtain restrictive and additive combinations, respectively. In addition, the search will be performed using the identified descriptors and with expanded meaning, without the use of database filters to preserve significant samples and ensure lower risk of loss. The search strategy combining the MeSH controlled descriptors and keywords that will be used in MEDLINE will be adjusted to the other electronic databases as described in Table 1.

**Table 1:** Preliminary search strategy in the MEDLINE via PubMed.

Database	Items searched
MEDLINE/PubMed	**#1** (“Home Care Services” [MeSH Terms] OR “Home Care Service” [Title/Abstract] OR “Service, Home Care” [Title/Abstract] OR “Services, Home Care” [Title/Abstract] OR “Care Services, Home” [Title/Abstract] OR “Domiciliary Care” [Title/Abstract] OR “Care, Domiciliary” [Title/Abstract] OR “Home Health Care” [Title/Abstract] OR “Home Care” [Title/Abstract] OR “Care, Home” [Title/Abstract])
	**#2** (“Hospital Costs” [MeSH Terms] OR “Cost, Hospital” [Title/Abstract] OR “Hospitals” [MeSH Terms] OR “Hospital” [Title/Abstract] OR “Hospital Units” [MeSH Terms] OR “Unit, Hospital” [Title/Abstract] OR “Inpatients” [MeSH Terms] OR “Inpatient” [Title/Abstract])
	**#3** (“Infant” [MeSH Terms] OR “Infants” [Title/Abstract] OR “Infant, Newborn” [MeSH Terms] OR “Infants, Newborn” [Title/Abstract] OR “Newborn Infant” [Title/Abstract] OR “Newborn Infants” [Title/Abstract] OR “Newborns” [Title/Abstract] OR “Newborn” [Title/Abstract] OR “Neonate” [Title/Abstract] OR “Neonates” [Title/Abstract] OR “Baby” [Title/Abstract] OR “Babies” [Title/Abstract] OR “Child, Preschool” [MeSH Terms] OR “Preschool Child” [Title/Abstract] OR “Children, Preschool” [Title/Abstract] OR “Preschool Children” [Title/Abstract] OR “Child” [MeSH Terms] OR “Children” OR “Adolescent” [MeSH Terms] OR “Adolescents” [Title/Abstract] OR “Adolescence” [Title/Abstract] OR “Teens” [Title/Abstract] OR “Teen” [Title/Abstract] OR “Teenagers” [Title/Abstract] OR “Teenager” [Title/Abstract] OR “Youth” [Title/Abstract] OR “Youths” [Title/Abstract])
	**#4** (#1 AND #2 AND #3)
	**#5** (“Cost-benefit Analysis” [MeSH Terms] OR “Cost-benefit” [Title/Abstract] OR “Cost benefit” [Title/Abstract] OR “Cost-effectiveness” [Title/Abstract] OR “Cost effectiveness” [Title/Abstract] OR “Cost-utility” [Title/Abstract] OR “Cost utility” [Title/Abstract] OR “Economic” [Title/Abstract] OR “Analysis” [Title/Abstract] OR “Evaluation” OR “Costs” [Title/Abstract] OR “Cost” [Title/Abstract])
	**#6** (#4 AND #5)

MEDLINE = medical literature analysis and retrieval system online.

In this search strategy phase the EndNote™ reference manager will be used to store, organize and delete duplicates to ensure a systematic, comprehensive and manageable search.

### 2.4. Eligibility

A summary of the inclusion and exclusion criteria of this systematic review is depicted in Table 2 in line with the acronym PECOS.

**Table 2:** Inclusion and exclusion criteria.

*PECOS acronym*	Inclusion criteria	Exclusion criteria
P—Population	Evidence from economic evaluations involving pediatric patients	Studies without cost, benefit and effectiveness analysis; studies involving adult and/or elderly patients
E—Exposure	Home Care Services	-
C—Comparison	Hospital care (in-hospital services)	-
O—Outcomes	Mortality, readmission rates, patient and caregiver satisfaction, and cost	-
S—Study Design	Observational or experimental studies of cost-effectiveness, cost-utility and cost-benefit evaluation	Qualitative studies

Regarding the study design, we will include analytical observational study designs and experimental studies, as well as gray literature, as recommended by the Cochrane Handbook.^[23]^ Thus, studies that investigated epidemiological and clinical aspects on the cost-effectiveness of HCS in pediatric patients (<19 years) in relation to in-hospital services will be included in this systematic review. Studies that do not present complete economic evaluations (evidence without cost, benefit and effectiveness analysis), and studies involving adult and/or elderly patients will be excluded. The selection of studies will also be performed by 2 reviewers independently (LCLJ and RMP) and blinded. After this selection, a third reviewer (RAGL) will be responsible for analyzing and deciding on the inclusion or exclusion of each article, especially those containing conflicting decisions. In this step of inclusion and exclusion of articles that will compose the final sample, the Rayyan™-Qatar CRI,^[26]^ as a tool to assist in the eligibility/selection of articles.

### 2.5. Data extraction

First, the screening of the studies will be based on the information contained in their titles and abstracts and will be performed by the same 2 researchers (LCLJ and RMP). When the reviewers disagree, the article will be evaluated, and if the disagreement persists, a third reviewer (RAGL) will make a final decision. Once consensus is reached on the selected studies, a specific standardized form for extracting previously published full economic evaluation studies will be used.^[6,27]^ The form is divided into 6 sections, according to the types of information made available by the studies: General information about the selected studies; Information about the study design, the population included and the comparators used; Information about the details of the economic model, time horizon and assumptions used and about the sensitivity analyses performed; Information about the costs; Sources of the data used in the study: epidemiological, intervention, effectiveness, cost and utility; Outcomes assessed in the studies and their measures and details of the sensitivity analysis.^[27]^

### 2.6. Methodological evaluation

The assessment of the methodological quality of the studies will be defined as an essential process to establish internal validity, checking for possible biases and reliability of the evidence identified. Initially, the level of evidence will be identified and classified according to the scale developed by the Oxford Centre for Evidence-Based Medicine that is based on study design and classifies them into 1A, 1B, 1C, 2A, 2B, 2C, 3A, 3B, 4, and 5 (Table 3).^[28]^

**Table 3:** Classification and hierarchy of evidence.

Level	Type of evidence
1A	Systematic review (with homogeneity) of RCTs
1B	Individual RCT (with narrow confidence intervals)
1C	All or none study
2A	Systematic review (with homogeneity) of cohort studies
2B	Individual Cohort study (including low quality RCT, e.g. <80% follow-up)
2C	“Outcomes” research; Ecological studies
3A	Systematic review (with homogeneity) of case-control studies
3B	Individual Case-control study
4	Case series (and poor quality cohort and case-control study
5	Expert opinion without explicit critical appraisal or based on physiology bench research or “first principles”

RCT = randomized controlled trials.

*From the Centre for Evidence-Based Medicine, http://www.cebm.net

Subsequently, the checklist for assessing the quality of reporting of economic evaluation studies proposed by The British Medical Journal will be applied^[29]^ that allows the evaluation of the items of an economic evaluation. This checklist is divided into 3 blocks of questions: study design; data collection; and analysis and interpretation of results. The internal validity and risk of bias of randomized controlled trials will be assessed using the Cochrane Risk-Of-Bias tool revised for randomized controlled trials.^[30]^ To assess the risk of bias in quasi-experimental studies, we will use the Risk of Bias In Non-randomized Studies of Interventions.^[31]^ In addition, the Newcastle-Ottawa Scale^[32]^ will be used to assess the internal validity and risk of bias of cohort studies. Cross-sectional studies will be evaluated using the Agency for Healthcare Research and Quality tool.^[33]^ The same 2 reviewers (LCLJ and RMP) will perform the critical appraisal independently. To assess the methodological quality of the comparative effectiveness observational research evidence we will use the Good Research for Comparative Effectiveness Checklist v5.0.^[34]^

### 2.7. Data analysis and evidence synthesis

Heterogeneity among studies will be measured by the *I*^2^ statistic to estimate the percentage of variation among studies, where *I*^2^ = 0% to 40% indicates low heterogeneity; *I*^2 ^= 30% to 60% moderate heterogeneity; *I*^2^ = 50% to 90% substantial heterogeneity; and *I*^2^ = 75% to 100% high heterogeneity.^[35,36]^ According to the *I*^2^ statistic, we will determine whether a meta-analysis is feasible. In this case, we will check the statistical model to be used to gather the study-specific estimates, i.e., fixed-effects model or random-effects model analysis.^[37–43]^ For data analysis, the calculation of pooled effect estimates will consider a CI = 95% and α = 0.05 using EPPI-Reviewer™ Software (UK). We will also assess publication bias if sufficient studies are identified per endpoint analyzed.^[44]^ In addition, we will rate the certainty of the evidence based on the Cochrane methods and according to Grading of Recommendations Assessment, Development and Evaluation.^[45]^ The evaluation of the quality of evidence will be performed independently and paired by 2 reviewers (LCLJ and EB). Disagreements will be handled by a 3rd reviewer (RAGL). All steps of this review will be conducted in EPPI-Reviewer™ Software (UK).

**Figure 1.**
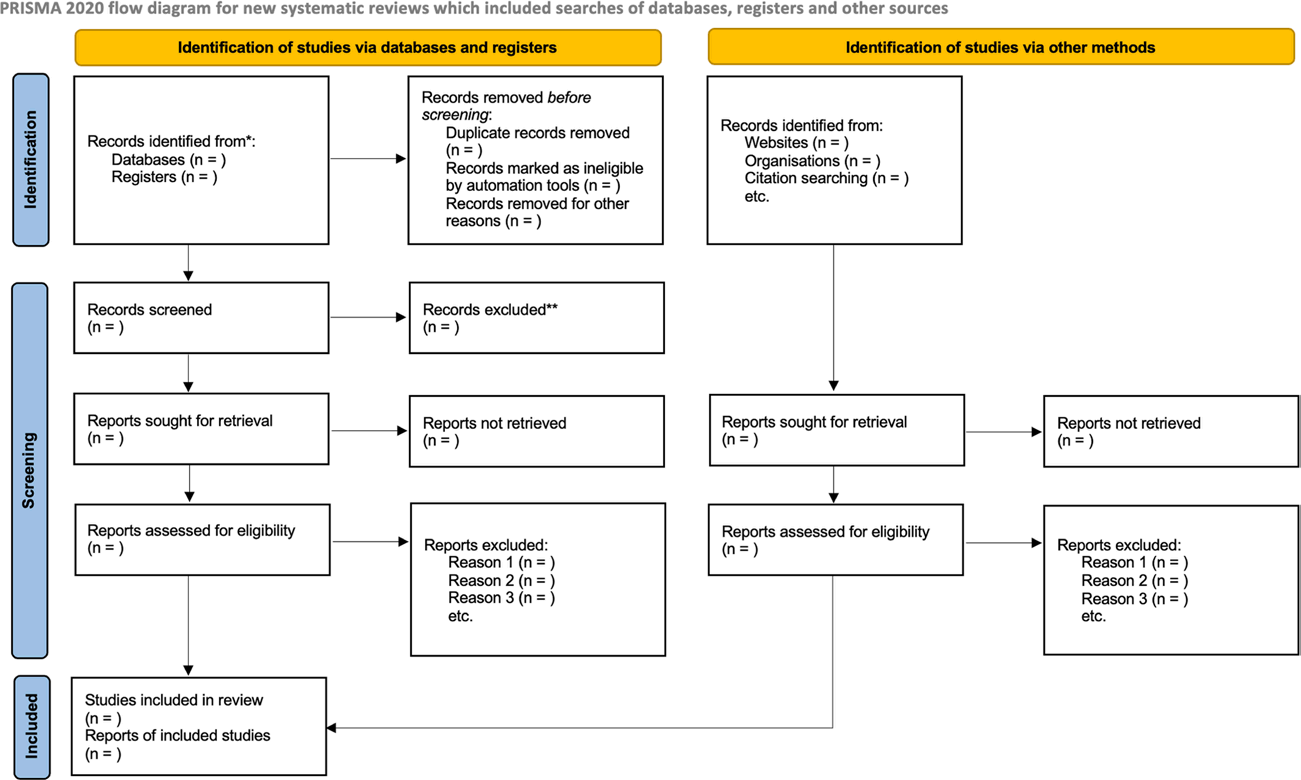
PRISMA flow diagram.

The study results will be presented in PRISMA 2020^[46]^ flowchart (Fig. 1), tables or graphs in the same way as the summaries are reported, in order to facilitate comparison of similarities and differences in the different study designs and outcomes between studies. The results will be presented and stratified in the subgroup analysis according to the health system models and in line with the income classification of the countries (high, medium-high, medium-low, and low) based on The World Bank Classification using the Gross National In-come per capita.^[47]^

### 2.8. Patient and public involvement

This study protocol analyses existing research studies, and therefore involves no patients or members of the public.

### 2.9. Ethics and dissemination

This study involves neither human participants nor unpublished primary data. As such, ethics approval from a human research ethics committee is not required. Plans for the dissemination of this study comprise peer-reviewed publication and conference presentations.

## 3. Discussion

The results of this research will bring systematic and with high methodological rigor the available evidence on the cost-effectiveness of home care in pediatric patients compared to in-hospital services, exploring different outcomes available in the literature in the various models of health systems in the world. These results may have relevance for the basis of public health policies, regarding the forms of organization of HCS, especially in terms of complete economic evaluations through cost-effectiveness analysis in relation to hospital care. It is expected that from this product, it will be possible to obtain the necessary elements to support decision making for changes in management, practice and training of health professionals, impacting on the quality of home care.

## Author contributions

LCLJ conceived the idea and planned and designed the study protocol. LCLJ wrote the first draft. LCLJ, RMP, EB and RAGL planned the data extraction and statistical analysis. LCLJ and RAGL provided critical insights. RMP and EB critically reviewed and modified the manuscript. All authors have reviewed and approved the manuscript. LCLJ is responsible for the overall content as guarantor.

**Conceptualization:** Luís Carlos Lopes-Júnior.

**Data curation:** Luís Carlos Lopes-Júnior, Emiliana Bomfim, Regina Aparecida Garcia de Lima.

**Formal analysis:** Luís Carlos Lopes-Júnior, Raphael Manhães Pessanha, Emiliana Bomfim, Regina Aparecida Garcia de Lima.

**Funding acquisition:** Luís Carlos Lopes-Júnior.

**Investigation:** Luís Carlos Lopes-Júnior.

**Methodology:** Luís Carlos Lopes-Júnior.

**Project administration:** Luís Carlos Lopes-Júnior.

**Resources:** Luís Carlos Lopes-Júnior.

**Software:** Luís Carlos Lopes-Júnior.

**Supervision:** Luís Carlos Lopes-Júnior.

**Validation:** Luís Carlos Lopes-Júnior, Raphael Manhães Pessanha, Emiliana Bomfim, Regina Aparecida Garcia de Lima.

**Visualization:** Luís Carlos Lopes-Júnior, Raphael Manhães Pessanha, Emiliana Bomfim, Regina Aparecida Garcia de Lima.

**Writing – original draft:** Luís Carlos Lopes-Júnior, Raphael Manhães Pessanha, Emiliana Bomfim, Regina Aparecida Garcia de Lima.

**Writing – review & editing:** Luís Carlos Lopes-Júnior, Raphael Manhães Pessanha, Emiliana Bomfim, Regina Aparecida Garcia de Lima.

## References

[1] Mendes EV. O cuidado das condições crônicas na atenção primária à saúde: o imperativo da consolidação da estratégia da saúde da família. Brasília: Organização Pan-Americana da Saúde. 2012.

[2] Rajão FL Martins M. Home care in Brazil: an exploratory study on the construction process and service use in the Brazilian health system. Cien Saude Colet. 2020;25:1863–77. 32402051 10.1590/1413-81232020255.34692019

[3] Braga PP de Sena RR Seixas CT. Supply and demand in home health care]. Cien Saude Colet. 2016;21:903–12. 26960102 10.1590/1413-81232015213.11382015

[4] Kerber NP Kirchhof AL Cezar-Vaz MR. Home care and its relationship to the work environment in health. Cad Saude Publica. 2008;24:485–93. 18327436 10.1590/s0102-311x2008000300002

[5] Loyola CMD. Cuidado continuado. Giovanella L Escore lS Lobato LVC Noronha JC Carvalho AI, orga-nizadores (eds). In: Políticas e Sistema de Saúde no Brasil. Rio de Janeiro: Editora Fiocruz; Centro Brasileiro de Estudos de Saúde. 2008:959–978.

[6] Brasil. Ministério da Saúde. Secretaria de Ciência, Tecnologia e Insumos Estratégicos. Departamento de Ciência e Tecnologia. Diretrizes metodológicas: Diretriz de Avaliação Econômica/ Ministério da Saúde, Secretaria de Ciência, Tecnologia e Insu- mos Estratégicos, Departamento de Ciência e Tecnologia. 2. ed. Brasília: Ministério da Saúde. 2014:132.

[7] Secoli SR Nita ME Ono-Nita SK. Health technology assessment: II. cost effectiveness analysis. Arq Gastroenterol. 2010;47:329–33. 21225140 10.1590/s0004-28032010000400002

[8] Moraz G Garcez Ada S de Assis EM. Cost-effectiveness in health in Brazil: a systematic review. Cien Saude Colet. 2015;20:3211–29. 26465862 10.1590/1413-812320152010.00962015

[9] Shepperd S Iliffe S Doll HA. Admission avoidance hospital at home. Cochrane Libr. 2016;9:CD007491. 10.1002/14651858.CD007491.pub2 PMC6457791 27583824

[10] Pouw MA Calf AH van Munster BC. Hospital at Home care for older patients with cognitive impairment: a protocol for a randomised controlled feasibility trial. BMJ Open. 2018;8:e020332. 10.1136/bmjopen-2017-020332 PMC5875621 29593022

[11] Genet N Boerma WGW Kringos DS. Home care in Europe: a systematic literature review. BMC Health Serv Res. 2011;11:207. 21878111 10.1186/1472-6963-11-207 PMC3170599

[12] Low LL Vasanwala FF Ng LB. Effectiveness of a transitional home care program in reducing acute hospital utilization: a quasi-experimental study. BMC Health Serv Res. 2015;15:100. 25888830 10.1186/s12913-015-0750-2 PMC4377016

[13] Voudris VK Silver AM. Home hospitalization for acute decompensated heart 31 failure: opportunities and strategies for improved health outcomes. Healthc. 2018;6:31. 10.3390/healthcare6020031 PMC6023525 29597247

[14] Szebehely M Trydegård G. Home care for older people in Sweden: a universal model in transition. Health Soc Care Community. 2012;20:300–9. 22141377 10.1111/j.1365-2524.2011.01046.x

[15] Rizzi M Grassi M Pecis M. A specific home care program improves the survival of patients with chronic obstructive pulmonary disease receiving long term oxygen therapy. Arch Phys Med Rehabil. 2009;90:395–401. 19254602 10.1016/j.apmr.2008.08.223

[16] Caplan GA Sulaiman NS Mangin DA. A meta-analysis of “hospital in the home”. Med J Aust. 2012;197:512–9. 23121588 10.5694/mja12.10480

[17] Parker G Spiers G Gridley K. Systematic review of international evidence on the effectiveness and costs of paediatric home care for children and young people who are ill. Child Care Health Dev. 2013;39:1–19. 22329427 10.1111/j.1365-2214.2011.01350.x

[18] Ramos MCP Barton P Jowett S. A systematic review of research guidelines in decision-analytic modeling. Value Heal. 2015;18:512–29. 10.1016/j.jval.2014.12.014 26091606

[19] Centre for Reviews and Dissemination. Universit of York. S stematic reviews: CRD’s guidance for undertaking reviews in health care [Internet]. York: York Publishing Services Ltd. 2009.

[20] Moher D Shamseer L Clarke M. PRISMA-P Group. Preferred reporting items for systematic review and meta-analysis protocols (PRISMA-P) 2015 statement. Syst Rev. 2015;4:1. 25554246 10.1186/2046-4053-4-1 PMC4320440

[21] Morgan RL Whaley P Thayer KA. Identifying the PECO: a framework for formulating good questions to explore the association of environmental and other exposures with health outcomes. Environ Int. 2018;121(Pt 1):1027–31. 30166065 10.1016/j.envint.2018.07.015 PMC6908441

[22] World Health Organization. Air pollution and child health: prescribing clean air. Geneva, Switzerland: World Health Organization, 2018.

[23] Higgins JPT Thomas J Chandler J. (eds). Cochrane Handbook for Systematic Reviews of Interventions version 6.2 (updated February 2021). Chichester, UK: Cochrane. 2021. Available at: www.training.cochrane.org/handbook [access date August 13, 2022].

[24] Lopes-Júnior LC Bomfim E Olson K. Effectiveness of hospital clowns for symptom management in paediatrics: systematic review of randomised and non-randomised controlled trials. BMJ. 2020;371:m4290. 33328164 10.1136/bmj.m4290 PMC7737653

[25] Lopes-Júnior LC Rosa MADRP Lima RAG. Psychological and psychiatric outcomes following PICU admission: a systematic review of cohort studies. Pediatr Crit Care Med. 2018;19:e58–67. 29189670 10.1097/PCC.0000000000001390

[26] Ouzzani M Hammady H Fedorowicz Z. Rayyan-a web and mobile app for systematic reviews. Syst Rev. 2016;5:210. 27919275 10.1186/s13643-016-0384-4 PMC5139140

[27] Salomon FCR. Revisão sistemática de estudos de avaliação econômica sobre o uso do brometo de tiotrópio para o tratamento da doença pulmonar obstrutiva crônica (Tese de Doutorado). Rio de Janeiro (RJ): FioCruz. 2013.

[28] Oxford Centre for Evidence-Based Medicine. Levels of evidence working group. “The Oxford 2011 Levels of Evidence”. Oxford centre for evidence-based medicine. Oxford, UK: Centre for Evidence-Based Medicine (CEBM), 2011.

[29] Drummond M Jefferson T. Guidelines for authors and peer reviewers of economic submissions to the BMJ. The BMJ economic evaluation working party. Br Med J. 1996;313:275–83. 8704542 10.1136/bmj.313.7052.275 PMC2351717

[30] Sterne JAC Savović J Page MJ. RoB 2: a revised tool for assessing risk of bias in randomized trials. BMJ. 2019;366:l4898. 31462531 10.1136/bmj.l4898

[31] Sterne JA Hernán MA Reeves BC. ROBINS-I: a tool for assessing risk of bias in non-randomised studies of interventions. BMJ. 2016;355:i4919. 27733354 10.1136/bmj.i4919 PMC5062054

[32] Wells GA Shea B O’Connell D. The Newcastle-Ottawa Scale (NOS) for assessing the quality if nonrandomized studies in meta-analyses. Available at: http://wwwohrica/programs/ clinical_epidemiology/oxfordasp [access date August 1, 2022].

[33] Methods guide for effectiveness and comparative effectiveness reviews [Internet]. Rockville (MD): Agency for Healthcare Research and Quality (US), 2008. 21433403

[34] Dreyer NA Schneeweiss S McNeil BJ. GRACE principles: recognizing high-quality observational studies of comparative effectiveness. Am J Manag Care. 2010;16:467–71. 20560690

[35] Higgins JPT Thompson SG. Quantifying heterogeneity in a meta-analysis. Stat Med. 2002;21:1539–58. 12111919 10.1002/sim.1186

[36] Higgins JPT Thompson SG Deeks JJ. Measuring inconsistency in metaanalyses. BMJ. 2003;327:557–60. 12958120 10.1136/bmj.327.7414.557 PMC192859

[37] Fokoua-Maxime CD Lontchi-Yimagou E Cheuffa-Karel TE. Prevalence of asymptomatic or “silent” myocardial ischemia in diabetic patients: protocol for a systematic review and meta-analysis. PLoS One. 2021;16:e0252511. 34111136 10.1371/journal.pone.0252511 PMC8191872

[38] Campbell M McKenzie JE Sowden A. Synthesis without meta-analysis (SWiM) in systematic reviews: reporting guideline. BMJ. 2020;368:l6890. 31948937 10.1136/bmj.l6890 PMC7190266

[39] Egger M Smith GD Schneider M. Bias in meta-analysis detected by a simple, graphical test. BMJ. 1997;315:629–34. 9310563 10.1136/bmj.315.7109.629 PMC2127453

[40] Lopes-Júnior LC Siqueira PC Maciel ELN. School reopening and risks accelerating the COVID-19 pandemic: a systematic review and meta-analysis protocol. PLoS One. 2021;16:e0260189. 34788344 10.1371/journal.pone.0260189 PMC8598030

[41] Silva Junior FJGD Sales JCES Monteiro CFS. Impact of COVID-19 pandemic on mental health of young people and adults: a systematic review protocol of observational studies. BMJ Open. 2020;10:e039426. 10.1136/bmjopen-2020-039426 PMC7358102 32611746

[42] Gonçalves CA Lopes-Júnior LC Nampo FK. Safety, efficacy and immunogenicity of therapeutic vaccines in the treatment of patients with high-grade cervical intraepithelial neoplasia associated with human papillomavirus: a systematic review protocol. BMJ Open. 2019;9:e026975. 10.1136/bmjopen-2018-026975 PMC6661674 31320349

[43] Pessanha RM Schuab SIPC Nunes KZ. Use of family history taking for hereditary neoplastic syndromes screening in primary health care: a systematic review protocol. PLoS One. 2022;17:e0271286. 35877607 10.1371/journal.pone.0271286 PMC9312395

[44] Duval S Tweedie R. Trim and fill: A simple funnel-plot-based method of testing and adjusting for publication bias in meta-analysis. Biometrics. 2000;56:455–63. 10877304 10.1111/j.0006-341x.2000.00455.x

[45] Balshem H Helfand M Schünemann HJ. GRADE guidelines: 3. Rating the quality of evidence. J Clin Epidemiol. 2011;64:401–6. 21208779 10.1016/j.jclinepi.2010.07.015

[46] Page MJ McKenzie JE Bossuyt PM. The PRISMA 2020 statement: an updated guideline for reporting systematic reviews. BMJ. 2021;372:n71. 33782057 10.1136/bmj.n71 PMC8005924

[47] The World Bank Classifying countries by income. IBRD. IDA. Available at: https://datatopics.worldbank.org/world-development-indicators/stories/the-classification-of-countries-by-income.html [access date July 30, 2022].

